# Development and Validation of a Reversed-Phase HPLC Method with UV Detection for the Determination of L-Dopa in *Vicia faba* L. Broad Beans

**DOI:** 10.3390/molecules27217468

**Published:** 2022-11-02

**Authors:** Carmen Tesoro, Rosanna Ciriello, Filomena Lelario, Angela Di Capua, Raffaella Pascale, Giuliana Bianco, Mario Dell’Agli, Stefano Piazza, Antonio Guerrieri, Laura Scrano, Sabino A. Bufo, Maria Assunta Acquavia

**Affiliations:** 1 Department of Sciences, University of Basilicata, Via dell’Ateneo Lucano 10, 85100 Potenza, Italy; 2 Gnosis Bioresearch by Lesaffre, 75015 Pisticci, Italy; 3 Department of Pharmacological and Biomolecular Sciences, University of Milan, Via Balzaretti 9, 20133 Milano, Italy; 4 Department of the European Cultures (DICEM), University of Basilicata, Via Lanera 20, 85100 Matera, Italy; 5 Department of Geography, Environmental Management & Energy Studies, University of Johannesburg, Johannesburg 2092, South Africa; 6 Thema Informatik S.R.L, Via Ressel 2/F, 39100 Bolzano, Italy

**Keywords:** drugs, bioactive compound, liquid chromatography, UV detection, broad beans, aqueous stability, EURACHEM guidelines, storage conditions

## Abstract

L-Dopa (LD), a substance used medically in the treatment of Parkinson’s disease, is found in several natural products, such as *Vicia faba* L., also known as broad beans. Due to its low chemical stability, LD analysis in plant matrices requires an appropriate optimization of the chosen analytical method to obtain reliable results. This work proposes an HPLC-UV method, validated according to EURACHEM guidelines as regards linearity, limits of detection and quantification, precision, accuracy, and matrix effect. The LD extraction was studied by evaluating its aqueous stability over 3 months. The best chromatographic conditions were found by systematically testing several C_18_ stationary phases and acidic mobile phases. In addition, the assessment of the best storage treatment of *Vicia faba* L. broad beans able to preserve a high LD content was performed. The best LD determination conditions include sun-drying storage, extraction in HCl 0.1 M, chromatographic separation with a Discovery C_18_ column, 250 × 4.6 mm, 5 µm particle size, and 99% formic acid 0.2% *v*/*v* and 1% methanol as the mobile phase. The optimized method proposed here overcomes the problems linked to LD stability and separation, thus contributing to the improvement of its analytical determination.

## 1. Introduction

**Figure 1:**
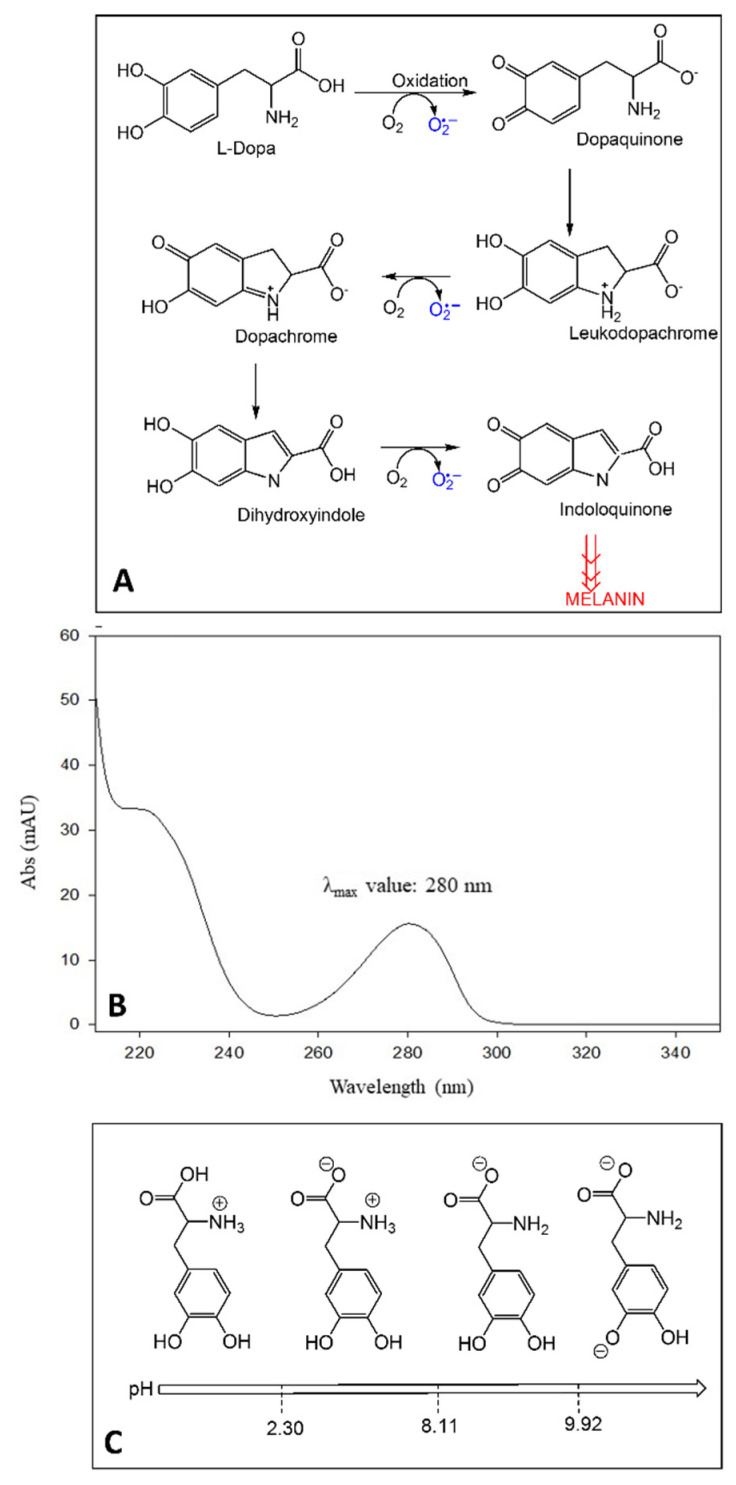
Auto-oxidation reactions of LD and ROS production (**A**); UV absorption spectrum of LD (**B**); LD structures at different pH and corresponding pKa values (**C**).

Broad bean (*Vicia faba* L.) has been identified as a rich source of L-Dopa or levodopa (LD), a dopamine precursor and first-line treatment for Parkinson’s disease (PD) symptoms, usually characterized by slowness of movement (bradykinesia), tremor at rest, muscle rigidity, and postural fragility [1,2,3,4,5,6]. PD affects nerve cells and their function in producing the neurotransmitter dopamine. In contrast to dopamine, LD can cross the blood-brain barrier and enter into the nerve cells, where it is decarboxylated to dopamine. In cells, LD can also be oxidized toward melanin, producing leucodopachrome and dopachrome by auto-oxidation or with the aid of tyrosinases, also known as polyphenol oxidases (PPO) (Figure 1A). During these reactions, reactive oxygen species (ROS) such as hydrogen peroxide (H_2_O_2_), superoxide anion (O_2_^•−^), and hydroxyl radical (HO^•^) can be produced. Some studies report that the beneficial effect of LD drugs in PD is counterbalanced by the strong oxidative damage generated over a long period of drug treatment [7]. Furthermore, patients with advanced PD generally experience an unbalanced response pattern to L-Dopa because of fluctuating drug delivery to the brain. In the most severe form, motor fluctuations produce the typical “on-off” syndrome. Thus, the consumption of vegetables containing LD, such as broad beans or botanicals food supplements, could be recommended as adjuvants for patients with PD [7,8]. In fact, as reported by Apaydin et al. [9]., the “on” period was prolonged in patients consuming a *Vicia faba* broad bean meal.

The presence of the catechol moiety in the chemical structure characterizes LD as a chromophore with a λ_max_ value of 280 nm and allows us to obtain UV detection results sufficiently sensitive for LD determination in vegetable matrices [10]. Since the absorption spectrum of LD is highly characteristic (Figure 1B), its determination by spectrum acquisition (LC-UV analysis) is also selective. Although several scientific works reported the separation and quantification of LD in *Vicia faba* L. by using liquid chromatography coupled to a UV–Vis detector (LC/UV–Vis) [8,10,11,12,13,14,15,16,17,18,19,20,21,22,23,24,25], there are still some limitations regarding LD extraction/stability, chromatographic conditions, and method validation.

Firstly, LD is unstable in aqueous solutions and naturally degrades over time [26,27,28,29,30,31,32,33,34,35,36]: for this reason, it is crucial to identify the best conditions to avoid LD degradation over all the analytical steps. LD instability will also influence the choice of the extraction technique. Typical extraction techniques include liquid–solid extraction, Soxhlet extraction, microwave-assisted extraction, and ultrasound-assisted extraction [10]. However, the latter two techniques, although significantly improving the extraction procedure in terms of automation and solvent consumption, are less used because thermal effects can reduce the final concentration of L-Dopa [11].

Secondly, low-molecular-weight polar compounds, such as LD, are generally difficult to separate by using reversed-phase liquid chromatography (RP-LC) [10]. Generally, it is required to work below the pKa of the compound where it will be completely protonated, or to use an ion pair reagent to increase retention time. Chromatographic conditions as regards LC column and mobile-phase composition are also fundamental to obtain reliable results. Mostly, C_18_ stationary phases have been used due to the highly polar structure of the analyte [10,11,13,23]. Acetate/phosphate buffers at acidic pH values (<3) have also been used, although some papers report the use of acetate/phosphate buffers at pH of 4.00, 4.55, 4.66, or 7.00 [10,19,20,23,24,37]. It should be pointed out that, under highly acidic conditions, both analyte and residual silanol groups on the silica column packing support are fully protonated, so they cannot interact electrostatically, avoiding tailing, peaks broadening, and poor retention reproducibility [37]. However, the use of a mobile phase with a certain percentage of organic phase should be considered to prevent the dewetting problem of the C_18_ stationary phase, and, as a consequence, to limit the lowering of its retention capacity [38,39]. For these reasons, an optimization of chromatographic conditions is necessary.

As no systematic LC-UV method validation has been reported in the literature to analyze LD occurring in *Vicia faba* L. broad beans, with just a few quality parameters of the chromatographic method being ascertained [16], it is crucial to validate a reliable LC-UV method for LD quantification in this matrix.

In this work, a validated LC-UV method for LD analysis according to EURACHEM guidelines has been proposed, alongside the study of its stability in different acidic solutions and the systematic optimization of chromatographic conditions as regards both chromatographic column and mobile phase. Then, the validated method was tested to evaluate the LD contents in seven *Vicia faba* L. broad bean samples, differently stored (fresh, sun-dried for 10 and 30 days, freeze-dried, frozen for 10 and 30 days, and commercial long-life frozen), to find the best storage conditions to limit LD degradation.

## 2. Results and Discussion

### 2.1. Aqueous Stability Study of LD

Several studies highlighted LD instability in solution and plant matrices [14,26,27,28,29,30,31,32]. Based on its chemical structure, LD has three ionizable groups (Figure 1C). When the pH is between 2.3 and 8.11, LD takes on a zwitterionic structure, which is involved in the intermolecular bond between the protonated amino group and the deprotonated carboxylic group, thus leading to aggregated structures, responsible for the lower LD solubility and stability in neutral environments [10,11,32]. Recent studies have also shown that even oxygen tension affects LD stability. Under normoxic conditions, the analyte is prone to auto-oxidation by releasing protons and reactive oxygen species (ROS) as intermediates [33,34]. In acidic conditions, protons supplied by solvents would shift the auto-oxidation reaction equilibrium towards the reagents and avoid the formation of intermolecular bonding networks [30,32]. Instead, alkaline pH values in plants were shown to increase the enzymatic activity of phenol oxidase (PO). By going from pH 3.5 to pH 4.5, PO activity rises from 60% to 100% [35], thus encouraging the employment of acidic environments when performing the determination of LD. Therefore, LD acidic structure requires working at a suitable mobile-phase pH value to avoid a significant conversion in the corresponding ionized structures [36].

**Figure 2:**
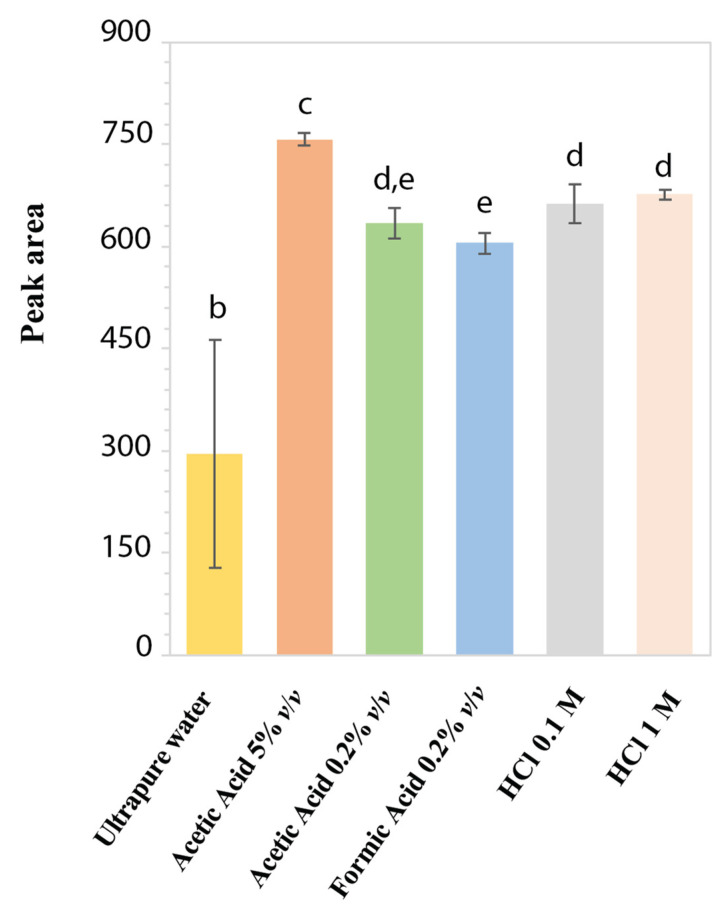
Stability histogram obtained by monitoring peak areas of LD standard solutions at 50 mg/L in different acidic media. Each bar represents the means ± standard deviation of the results obtained weekly for 3 consecutive months. Values marked by the same letter are not significantly different (*p* < 0.05).

To assess the aqueous stability and to identify the best extraction solvent, the performance of different standard acidic solutions of LD at 50 mg/L was evaluated in terms of reproducibility of chromatographic peak area. In Figure 2, the stability histogram of LD peak area is reported as mean values over 3 months with the corresponding standard deviations. Since the LD solution in ultrapure H_2_O presented the lowest mean peak area and the highest standard deviation because of the area decreasing over time, it was ascertained as the least stable compared to the other acidic solutions. Furthermore, after two weeks, the solution acquired a dark color due to the formation of melanin [29]: this result confirmed the LD degradation and its chemical instability in H_2_O, as already reported for LD solutions with alkaline pH or close to neutrality [10,11,29,32]. Instead, for standard LD solutions at acidic pH, the chromatographic peak area values remained stable over 3 months, with %RSD ranging from 0.99% to 4.30%. The best reproducibility in chromatographic peak areas was obtained with standard solutions in HCl 1 M (0.99% RSD) and acetic acid 5% *v*/*v* (1.17% RSD). However, in HCl 1 M, an additional chromatographic peak was observed rising over time, probably due to hydrolysis of LD. On the other hand, LD in acetic acid at 5% *v*/*v* showed a UV-absorption spectrum completely different from the characteristic profile of LD, reported in Figure 1A. This could be related to a possible analyte acetylation due to the presence of acetic acid at a high concentration. Based on these results, HCl 0.1 M was selected to prepare LD standard solutions as an appreciable signal intensity, with a good reproducibility (4.30% RSD) of the chromatographic peak areas was obtained [9].

### 2.2. Chromatographic Performances

In the present work, a careful investigation of the LD separative chromatographic conditions has been carried out to develop a suitable method for analyzing *Vicia faba* L. extracted samples.

#### 2.2.1. Choice of the Chromatographic Column

**Figure 3:**
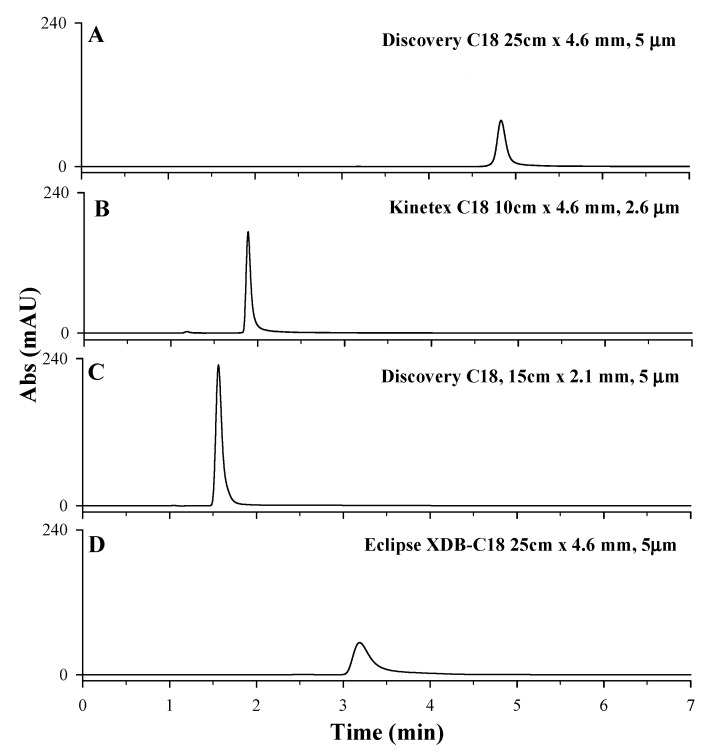
HPLC-UV chromatographic profiles obtained during the optimization of stationary phase: (**A**) Discovery C_18_ column, 250 × 4.6 mm, 5 µm; (**B**) Kinetex C_18_ column, 100 × 4.6 mm, 2.6 µm particle size; (**C**) Discovery C_18_ column, 150 × 2.1 mm, 5 µm; (**D**) Agilent ZORBAX Eclipse XDB C_18_ column, 250 × 4.6 mm, 5 µm. An LD standard solution at 50 mg/L solubilized in HCl 0.1 M, 97% acetic acid 0.2% *v*/*v*, and 3% methanol as mobile phase, under isocratic conditions depending on the tested C_18_ column (1 mL/min for Discovery C18 column, 0.8 mL/min for Kinetex C18 column and Agilent ZORBAX Eclipse XDB, 0.5 mL/min for Discovery Supelco C18 column.), injection volume of 20 µL and λ_max_ set of 280 nm, were used.

Starting from the chromatographic method proposed by Polanowska et al. [11], the performances of four different reverse-phase chromatographic columns (Agilent ZORBAX Eclipse XDB, Kinetex C_18_ column, 100 × 4.6 mm, 2.6 µm, Discovery C_18_ column, 150 × 2.1 mm, 5 µm, Discovery C_18_ column, 250 × 4.6 mm, 5 µm) have been tested (for columns characteristics see Section 3). Owing to its high and strong retention towards polar compounds, the porous graphitic carbon (PGC) analytical column is not the best choice for trapping very polar compounds at highly aqueous conditions and, for this reason, it was not considered in this study [40]. As reported in Figure 3, LD retention times and peak shapes changed according to the analytical chromatographic column used. The Agilent ZORBAX Eclipse XDB column was successfully used by Long et al. [36] for the separation of acids, bases, and other highly polar analytes in reversed-phase liquid chromatography. However, when used for LD separation, the HPLC-UV chromatographic profile showed a broad and asymmetric chromatographic peak; hence, the column is not suitable for the analysis (Figure 3D). Kinetex C_18_ column, 100 × 4.6 mm, 2.6 µm particle size, and Discovery C_18_ column, 150 × 2.1 mm, 5 µm particle size showed the highest intensity of chromatographic peaks but poor retention of LD analytes, with retention times lower than 2 min, too close to the solvent front (Figure 3B,C). Discovery C_18_ column, 250 × 4.6 mm, 5 µm particle size, instead showed an LD symmetrical peak eluted at 4.8 min. (Figure 3A) [10]. Although chromatograms in plots A and D were obtain with two columns with the same length, internal diameter (ID), and particle sizes, peaks show different retention time. This could be related to the different chemical and physical properties of the stationary phase, such as the type and surface area. In fact, the surface area accessible in a column can be considered as a key factor in influencing the retention of the analyte on different columns with the same type of stationary phase. The higher surface area of the Discovery C_18_ provides a greater number of binding sites compared to the Eclipse XDB (see Section 3), increasing the retention of the analyte. Based on these results, Discovery C_18_ column, 250 × 4.6 mm, 5 µm particle size was considered the most suitable for LD separation.

#### 2.2.2. Optimization of the Mobile-Phase Composition

**Figure 4:**
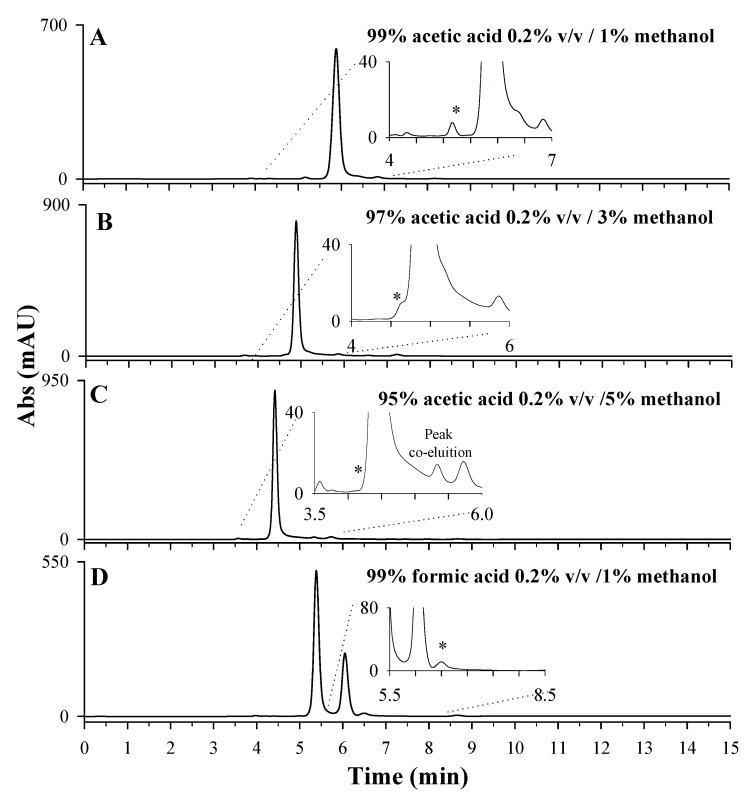
HPLC-UV chromatographic profiles obtained during the optimization of mobile-phase composition: 99% acetic acid 0.2% *v*/*v* containing 1% methanol (pH 3.04) (**A**); 97% acetic acid 0.2% *v*/*v* containing 3% methanol (pH 3.13) (**B**); 95% acetic acid 0.2% *v*/*v* containing 5% methanol (pH 3.13) (**C**); 99% formic acid 0.2% *v*/*v* containing 1% methanol (pH 2.61) (**D**). The LD peak is indicated with *. A 10-day dried extract of *Vicia faba* L. sample, diluted 1:10 in HCl 0.1 M, Discovery C_18_ column, 250 × 4.6 mm, 5 µm, under isocratic conditions (flow rate 0.1 mL/min), injection volume of 20 µL and λ_max_ set of 280 nm, were used.

After choosing the most suitable stationary phase for LD separation, the mobile phase’s optimization tests were carried out on the sample extracts of *Vicia faba* L. broad beans to evaluate potential interferences. Four different mobile phases were tested, as reported in Figure 4: 99% of acetic acid 0.2% *v*/*v* containing 1% of methanol (Figure 4A); 97% of acetic acid 0.2% *v*/*v* containing 3% of methanol (Figure 4B); 95% of acetic acid 0.2% *v*/*v* containing 5% of methanol (Figure 4C); 99% of formic acid 0.2% *v*/*v* containing 1% of methanol (Figure 4D). A percentage of methanolic organic phase not higher than 5% was used to limit the collapse of the stationary phase. By comparing plots A, B, and C, it is possible to observe a clear improvement in terms of resolution of the LD chromatographic peak, due to an increase in percentage of the aqueous phase, containing 0.2% acetic acid, from 95% (Figure 4C) to 99% (Figure 4A). A similar trend was observed for 0.2% *v*/*v* formic acid as the aqueous phase. Having set the aqueous-phase percentage of 99%, the performances of the two acids, formic and acetic, were compared (Figure 4A,D). In both cases, a well resolved peak was observed for LD (see insets) even if a chromatographic separation of the most abundant interference compounds is achieved only using formic acid (Figure 4D). Furthermore, the employment of formic acid ensured a longer retention time for LD (6.5 min) compared to acetic acid (5.2 min). Formic acid is known to be an ion-pairing reagent, whereas acetic acid is not. The authors of [36] state that ionic interactions between the formate group (-COO^−^) and basic group (-NH_3_^+^) of LD occur and are responsible for the increase in retention time, improving the selectivity of chromatographic separation [37].

After choosing 99% formic acid 0.2% *v*/*v*/1% methanol as the mobile phase, a new set of chromatographic runs of *Vicia faba* L. extracts were carried out on the Discovery C_18_ column, in order to compare the selected mobile phase with the 100% phosphate buffer 125 mM (pH 2.5) [41]. In fact, a buffered mobile phase is recommended in the literature to guarantee a stable pH system value for good peak shape and retention time reproducibility.

The use of phosphate buffer as the mobile phase improved the chromatographic separation efficiency but, over time, caused blockages to the pump and pressure drops due to the precipitation of phosphate salts at the LC valves, requiring additional cleaning of the system to ensure reproducibility and repeatability. Furthermore, considering the higher compatibility of formic acid with most powerful detection techniques such as mass spectrometry, the use of 99% of formic acid 0.2% *v*/*v* containing 1% of methanol is the most suitable choice as the mobile phase.

In conclusion, the optimal chromatographic conditions for LD separation in plant matrices of *Vicia faba* L. can be achieved by using the Discovery C_18_ column, 250 × 4.6 mm, 5 µm particle size as a stationary phase and a mobile phase composed of 99% formic acid 0.2% *v*/*v* containing 1% methanol, under isocratic conditions with a flow of 1 mL/min.

### 2.3. Method Validation

The performances of the analytic method were evaluated in terms of the estimation of linearity, LOD (limit of detection), LOQ (limit of quantification), accuracy, precision, matrix effect, and measurement uncertainties, according to European Action in Chemistry guidelines [42,43]. All these parameters are reported in Table 1.

The proposed method showed significant accuracy, precision, and linearity in the concentration range 0.5–50 mg/L. This range is much lower in concentration than that reported in the literature for *Vicia faba* L. matrix by Vora et al. [16], which validated a linear range 100–700 mg/L by using the same detector.

The calculated LOD and LOQ values of 0.0414 mg/L and 0.0452 mg/L, respectively, were lower than those of the only validated method by LC-UV reported in the literature for *Vicia faba* L. [16], highlighting the improved suitability of the proposed method for determination in samples with low LD content.

As regards the matrix effect (ME), a value of 100% means that there is not any kind of ME, as obtained in the proposed work. Therefore, it is possible to assess that the presence of matrix components does not interfere with the analysis of LD over the validated range.

The uncertainty measurements were expressed as expanded uncertainty (U, mg/L) for a normal distribution at a 95% confidence level [43,44], and the obtained results are reported in Table 1.

Finally, the results of tailing factor (T) and resolution (Rs) of the method at the three concentration levels, reported in Table 1, met the requirements for a good chromatographic method, showing well resolved peaks (R > 1.5) and no back tailing [45]

### 2.4. Quantitative Analysis of LD in Differently Stored Samples of Vicia faba L.

The proposed LC-UV method was successfully applied for the quantitative determination of LD occurring in local *Vicia faba* L. broad beans. Extracts from fresh broad bean sample were firstly analyzed. Then, the same sample was subjected to different storage treatments: sun-drying, freezing, and freeze-drying. Drying and freezing times were also varied (10 and 30 days). The aim was to suggest the best conditions able to preserve the LD content found in fresh samples. Table 2 shows the LD content in mg/g dry weight (dw) occurring in fresh sample, 10-day sun-dried sample, 30-day sun-dried sample, freeze-dried sample, 10-day frozen sample, 30-day frozen sample, and commercial long-life frozen sample.

The most abundant LD concentration was detected for 10-day sun-dried sample (1.26 ± 0.15 mg/g dw), which was not significantly different from the content occurring in the fresh sample (1.21 ± 0.17 mg/g dw). Interestingly, the LD content decreased by prolonging sun-drying up to 30 days. Freezing proved to be a less effective conservation method since after 10 days, LD content was already reduced to 1.03 ± 0.14 mg/g dw and after 30 days, it further decreased. For the extreme situation of indefinitely prolonged freezing time, as the commercial long-life frozen sample, LD content decreases so much as to become undetectable using the proposed HPLC-UV method. The freeze-drying storage preserved the LD content better than the 30-day freezing treatment. Furthermore, for samples with a short storage time (10 days), a difference between the freezing and drying treatments was observed. Compared to freezing storage, it seems that the conservation of broad beans by sun-drying allows for better preservation of the analyte, in agreement with the literature data [46,47]. Some papers highlight that sudden changes in temperature cause inhibition of the plant enzymatic activity of cytochrome P450, responsible for the synthesis of LD. Additionally, LD instability under strong thermal processes was largely demonstrated (e.g., freeze-drying, freezing, cooking, and high-temperature heating) [11,23,31,48].

Therefore, this study confirmed the convenience of using short-time sun-dried samples, which guarantee high and reproducible LD contents.

## 3. Materials and Methods

### 3.1. Chemicals

Methanol (≥99.8%) was purchased from Honeywell (Seelze, Germany). Analytical standard (≥98%) of 3,4-Dihydroxy-L-phenylalanine (LD) was purchased from Sigma-Aldrich (Milano, Italy). Formic acid was purchased from Fluka (Buchs, Switzerland). Potassium dihydrogen phosphate (≥98%), acetic acid (≥96%) were acquired from Carlo Erba (Rodano, Italy). Hydrochloric acid 37% (GR for analysis) was purchased from Merck KGaA (Darmastadt, Germany). Ultrapure water was produced using a Milli-Q RG system from Millipore (Bedford, MA, USA).

### 3.2. LD Stability Study

Stock solutions of LD were prepared at 1000 mg/L, in different acidic solutions: acetic acid 0.2% *v*/*v* (pH 3.29), formic acid 0.2% *v*/*v* (pH 2.62), acetic acid 5% *v*/*v* (pH 2.40), HCl 1 mol/L (pH 0), HCl 0.1 mol/L (pH 1), and ultrapure H_2_O (pH 5.37). LD working standard solutions at 50 mg/L were prepared by dilution from stock solutions and injected weekly by using Discovery C_18_ column, 250 × 4.6 mm, 5 µm particle size and the chromatographic conditions proposed by Polanowska et al. [11]. This study was performed for 3 months to monitor the analyte stability in the aqueous solutions over a long time. All solutions were stored at 4 °C in the dark to minimize thermal and photolytic degradation [32]. Student’s *t*-test (SPSS 19.0 for Windows; IBM SPSS Statistics, Armonk, NY, USA) was used to assess the presence of significant differences (*p* < 0.05) among the standard solution.

### 3.3. Analytical Method Validation

To optimize the method for the determination of LD by LC-UV system, the following parameters were assessed: LC performance, i.e., C_18_ stationary phases and mobile phases, and validation parameters, i.e., linearity, limit of detection (LOD), limit of quantification (LOQ), precision, accuracy, matrix effects, and uncertainties, according to European Action in Chemistry (EURACHEM) guidelines [35].

#### 3.3.1. LC Performance

Experiments were performed by using an Agilent 1200 Series Gradient HPLC System (Agilent Technologies, Santa Clara, CA, USA) equipped with a quaternary gradient pump unit, a diode array detector (DAD, 190–950 nm), and a standard autosampler (0.1 μL–100 μL) set to inject 20 μL. For all samples, to improve method selectivity, chromatograms at λ = 280 nm, combined with the absorbance spectrum (190–400 nm) were acquired and purity of LD peaks was checked. All the experiments were carried out at room temperature (25 °C).

Four different C_18_ analytical columns (Table 3) were used to optimize the best chromatographic separation: Discovery C_18_ column, 250 × 4.6 mm, 5 µm particle size; Kinetex C_18_ column, 100 × 4.6 mm, 2.6 µm particle size; Discovery Supelco C_18_ column, 150 × 2.1 mm, 5 µm particle size; ZORBAX Eclipse XDB-C_18_, 150 × 4.6 mm, 5 µm particle size. For the stationary-phase optimization study, a standard LD solution at 50 mg/L in 0.1 M HCl was injected, and chromatographic runs were carried out by using the mobile phase proposed by Polanowska et al. [11]: 97% of acetic acid at 0.2% *v*/*v* as aqueous phase containing 3% of methanol as organic phase, under isocratic conditions at 1 mL/min for Discovery C_18_ column, 0.8 mL/min for Kinetex C_18_ column and Agilent ZORBAX Eclipse XDB, 0.5 mL/min for Discovery Supelco C_18_ column.

Four different mobile phases were tested on the chosen column: 99% acetic acid 0.2% *v*/*v* (aqueous phase) containing 1% methanol (organic phase); 97% acetic acid 0.2% *v*/*v* (aqueous phase) containing 3% methanol (organic phase); 95% acetic acid 0.2% *v*/*v* (aqueous phase) containing 5% methanol (organic phase); 99% formic acid 0.2% *v*/*v* (aqueous phase) containing 1% methanol (organic phase). A 100% aqueous phase A consisting of a KH_2_PO_4_ 125 mM buffer solution (adjusted to pH 2.5 with concentrated H_3_PO_4_) was also tried. For mobile-phase optimization, 10-day sun-dried extract of *Vicia faba* L. sample, diluted 1:10 in HCl 0.1 M, Discovery C_18_ column, 250 × 4.6 mm, 5 µm, under isocratic conditions (flow rate 1 mL/min) was used.

All tests were performed in triplicate. Data acquisition and analyses were accomplished using the HPLC 1200 offline (Agilent Technologies, Santa Clara, CA, USA). The chromatographic raw data were imported, elaborated, and plotted by SigmaPlot 11.0 (Systat Software, Inc., London, UK).

#### 3.3.2. Validation Parameters

The linearity was assessed by the least-squares method in a concentration range between 0.5–50 mg/L of LD working solutions in 0.1M HCl. The linearity parameter was estimated at six concentration levels (*k* = 6) and the analyses were performed in three independent replicates (*n* = 3). Additionally, a statistic *t*-test was performed in order to assess the significativity of the correlation coefficient *R^2^* [43,44,49,50].

LOD and LOQ were evaluated by analyzing ten independent blank samples, calculating the mean blank response (x_b_) and its standard deviation (s_b_) as follows: yLoD = x_b_ + 3s_b_ and yLoQ = x_b_ + 10s_b_.

The precision of the proposed method was studied as “repeatability” and “intermediate precision”, expressed as percentage relative standard deviation (%RSD). The first parameter is the precision under the same operating conditions over a short time interval, i.e., the %RSD for six replicates (*n* = 6) of three levels (*k* = 3) over the linear range in the same day (*p* = 1). The second is the within-laboratories variations (different days, different analysts, different equipment, etc.) [51], i.e., the %RSD within several days (*p* = 3) for the ten replicates (*n* = 10) of three levels (*k* = 3) over the linear range. Furthermore, according to the EURACHEM guidelines, the repeatability limit and the intermediate repeatability limit were also calculated as follows: r = √2 × t_crit_ × s, where s is the standard deviation obtained under repeatability (*n* = 6) and intermediate precision (*n* = 10), the factor √2 reflects the difference between the two measurements, and the t_crit_ value is chosen at a 95% confidence level using a two-tailed distribution and a number of degrees of freedom equal to (*n*−1).

Since a certified reference material is not commercially available for the plant matrix under study, the accuracy, expressed as recovery, was evaluated by fortifying the real samples at least at three concentration levels (*k* = 3) over the linear range for three replicates (*n* = 3) at each level, over three days (*p* = 3). Since the long-term dried commercial broad bean sample had an LD quantity significantly lower than the LOD and LOQ values of the proposed method, it was used for recovery evaluation. The fortified LD concentration levels were: 0.5 mg/L (5 mg/Kg), 15 mg/L (150 mg/kg), and 50 mg/L (500 mg/kg). The fortified broad bean samples and the corresponding blank samples without fortification were analyzed to calculate the concentration of the analyte from the calibration curve. The difference between the analyte amount in spiked (C_sample_spk_) and unspiked (C_sample_unspk_) samples was divided by the amount of spike (C_spk_), to estimate the recovery as follows [42,43,49]: R_m_ = (C_sample_spk_ − C_sample_unspk_)/(C_spk_). The recovery standard deviation (U(R_m_)) was calculated by following this equation [44,52]:

(1) $$U(R_{m})=R_{m}\times \sqrt{\frac{\frac{s_{\mathrm{sample}\_\mathrm{spk}}^{2}}{n}+{\;s}_{\mathrm{sample}\_\mathrm{unspk}}^{2}}{{\left(C_{\mathrm{sample}\_\mathrm{spk}\;}-{\;C}_{\mathrm{sample}\_\mathrm{unspk}}\right)}^{2}}\;{\left(\frac{s_{\mathrm{spk}}}{C_{\mathrm{spk}}}\right)}^{2}}$$

where s_sample_spk_ and s_sample_unspk_ are the standard deviations of LD occurring in spiked and unspiked samples, and s_spk_ is the spike uncertainty.

Matrix effects (ME) were assessed using the post-extraction additions, which involve the calibration curve preparation with real extracts. Therefore, the calibration curve of the real samples was compared to the one achieved for the same standards in HCl 0.1 mol/L solvent. If both curves were parallel and overlapped, compounds are not subjected to any matrix effects. The ME was estimated by dividing the slopes of the matrix-matched calibration curves prepared with real extracts (slope matrix) and the slopes of the calibration curves prepared with solvent (slope std): ME(%) = 100 × (slope_matrix_/slope_std_) [43].

Finally, the expanded uncertainty was estimated as a combination of different contributions by using the bottom-up approach [43,44,52], as follows:

(2) $$u_{c}/C_{0}=\sqrt{{\left(\frac{u_{\mathrm{prep}}}{C_{0}}\right)}^{2}+{\left(\frac{u_{\mathrm{cal}}}{C_{0}}\right)}^{2}+{\left(\frac{u_{\mathrm{Rm}}}{\mathrm{Rm}}\right)}^{2}+{\left(\frac{u_{\mathrm{LoD}}}{\mathrm{LoD}}\right)}^{2}}$$

where u_c_ is the combined standard uncertainty (mg/L); C_0_ is the concentration level (mg/L); R_m_ is the recovery (%); LOD is the limit of detection (mg/L); u_prep_ is the uncertainty related to concentration levels preparation (mg/L); u_cal_ is the uncertainty of calibration curves (mg/L); u_Rm_ is the recovery uncertainty; u_LoD_ is the LoD uncertainty. Finally, extended uncertainties U, for *n* = 3 replicates, was estimated by multiplying the compound uncertainty by a coverage factor corresponding to *k* = 1.98, for a confidence level of 95%.

### 3.4. Vicia faba L. Broad Beans and LD Extraction

*Vicia faba* L. broad beans were purchased from a local producer in Potenza (Basilicata, Italy) as fresh sample. *Vicia faba* L. broad beans were then divided in different aliquots for different storage treatments: sun-drying for 10 and 30 days, freezing for 10 and 30 days, freeze-drying. Extraction conditions proposed by Polanowska et al. [11] were optimized to allow LD extraction from the fresh sample, sun-dried samples (10 and 30 days), freeze-dried sample, frozen samples (10 and 30 days), and commercial long-life frozen sample. Briefly, ultrasonic assisted extraction (UAE) was applied by using an extraction ratio of 1:10 weight/dry volume and HCl 0.1 mol/L as extracting solution; a sonication time of 20 min in an ice bath (4 °C) and a centrifugation for 10 min at 6000× *g* was applied. This procedure was performed twice, and then supernatants were collected, filtered on PTFE 0.2 µm filters, and stored at 4 °C in the dark until the LC-UV analyses. The LD quantification on the various broad bean extracts was performed by the external standard method. A botanical sample is kept in the Science Department of the University of Basilicata. The genus and species of the plant have been unambiguously identified.

## 4. Conclusions

An LC-UV method has been successfully optimized and validated for L-Dopa separation and quantification in *Vicia faba* L. broad bean samples. A strongly acidic aqueous solution, consisting of HCl 0.1 M, proved to be the best extraction solvent to assure the stability of LD over 3 months. After testing different stationary and mobile phases, a Discovery C_18_ column, 250 × 4.6 mm, 5 µm particle size as a stationary phase and a mobile phase consisting of 99% formic acid 0.2% *v*/*v* and 1% methanol (pH 2.61), under isocratic flow of 1 mL/min, were chosen for a reliable chromatographic separation. A rigorous LC-UV method validation, according to EURACHEM guidelines, reached LOD and LOQ values of 0.0414 and 0.0452 mg/L, respectively. High precision (less than 6.87% RSD) and accuracy (ranging between 1.01 and 1.03 of recovery) were obtained. No matrix effect was detected for the samples under study.

After validation, the proposed method was used for the LD quantitative analysis of differently stored *Vicia faba* L. broad bean samples, thus defining 10-day sun-drying as the best storage treatment able to preserve a high LD content in broad beans (1.26 ± 0.15 mg/g dw). The method described in this work was demonstrated to be robust and reliable for routine LD analyses in vegetable matrices, such as *Vicia faba* L. broad beans, which could be a potential functional food or an ingredient for food supplement preparation for PD patients.

## Figures and Tables

**Table 1:** Calibration curves, limit of detection and limit of quantification, precision, recovery, uncertainties, resolution, and tailing factor for LD determination by using LC-UV method.

Calibration Curve		Precision						Chromatographic Parameters	
Linear Range (*t* calc.) ^a^	Linear Equation ^b^, *R*^2^	LoD (mg/L) LoQ (mg/L)	Level (mg/L)	Rep. RDS% (*r* calc.) ^c^	Int. Precision RDS% (*r* calc.) ^d^	Recovery, R_m_ ± u(R_m_)	U (mg/L)	Rs ^e^	T ^e^
0.5–50 mg/L (99.98)	y = (13.99 ± 0.15) × + (6.35 ± 3.52) *R*^2^ = 0.999	0.0414 0.0452	0.5 15 50	1.41 (0.36) 0.16 (1.31) 0.11 (2.73)	6.87 (1.44) 2.57 (17.80) 2.20 (48.38)	1.01 ± 0.02 1.171 ± 0.002 1.033 ± 0.004	0.24 0.93 2.93	1.64 ± 0.03 1.65 ± 0.02 1.67 ± 0.03	1.14 ± 0.02 1.13 ± 0.01 1.13 ± 0.01

^a^ Calculated *t* value was compared to tabulated value *t*_0.01,4_ = 4.60 (*k* = 6). ^b^ Calibration fitting: y = x (m ± s_m_) + q ± s_q_. ^c^ The repeatability was estimated for six replicates (*n* = 6) of three levels (*k* = 3) over the linear range in the same day (*p* = 1) and *r* was calculated according to tabulated value *t*_0.05,5_ = 2.57. ^d^ The intermediate precision was calculated within 3 days (*p* = 3) for the ten replicates (*n* = 10) of three levels (*k* = 3) over the linear range and *r* was calculated according to tabulated value *t*_0.05,5_ = 2.26. ^e^ Chromatographic parameter were estimated as mean values ± SD (standard deviations).

**Table 2:** LD quantification in seven *Vicia faba* L. broad beans, stored by different processes: fresh sample, 10-day sun-dried sample, 30-day sun-dried sample, freeze-dried sample, 10-day frozen sample, 30-day frozen sample, and commercial long-life frozen sample. LD content is expressed as (mg/g dw) ± U(mg/g). Values marked by the same letter are not significantly different (*p* < 0.05).

Conservation Type	LD Concentration (mg/g dw)
*Vicia faba* L. fresh samples	1.21 ± 0.17 (a,b)
*Vicia faba* L. dried in the sun for 10 days	1.26 ± 0.15 (c,b)
*Vicia faba* L. dried in the sun for 30 days	0.81 ± 0.11 (f,g)
*Vicia faba* L. freeze-dried	0.76 ± 0.11 (h,g)
*Vicia faba* L. frozen for 10 days	1.03 ± 0.14 (d)
*Vicia faba* L. frozen for 30 days	0.51 ± 0.08 (e)
*Vicia faba* L. commercial long-life frozen	<LOQ

**Table 3:** Characteristics of columns tested in this work.

Column	Column Length (cm)	Column ID (mm)	Particle Size (µm)	Pore Size (Å)	Surface Area (m^2^/g)	Carbon Content (%)	pH Range	Matrix Functional Group	Supplier
Discovery^®^ C_18_ HPLC Column	15 cm	2.1 mm	5 μm	180 Å	300 m^2^/g	12%	2–8	C_18_ (octadecyl) phase endcapping	Supelco
Discovery^®^ C_18_ HPLC Column	25 cm	4.6 mm	5 μm	180 Å	300 m^2^/g	12%	2–8	C_18_ (octadecyl) phase endcapping	Supelco
Kinetex Core-Shell Column	10 cm	4.6 mm	2.6 μm	100 Å	200 m^2^/g	12%	1.5–10	C_18_ with TMS endcapping	Phenomenex
Eclipse XDB-C_18_ Column	25 cm	4.6 mm	5 μm	80 Å	180 m^2^/g	10%	2–9	dimethyl-n-octadecylsilanes double endcapping	Agilent

## Data Availability

Not applicable.

## Sample Availability

Samples of the compounds are available from the authors.
